# Supplementary material for: A connection between Vibrio cholerae motility and inter-animal transmission
Source: bioRxiv. 2025 Feb 13:2025.02.12.637895. Preprint. [Version 1] doi: 10.1101/2025.02.12.637895 (PMC11844489; doi:10.1101/2025.02.12.637895)
Supplement: 1 [file NIHPP2025.02.12.637895V1-supplement-1.pdf]

bioRxiv preprint doi: https://doi.org/10.1101/2025.02.12.637895; this version posted February 13, 2025. The copyright holder for this preprint (which was not certified by peer review) is the author/funder, who has granted bioRxiv a license to display the preprint in perpetuity. It is made available under aCC-BY 4.0 International license.

443 ## Supplemental Figures

444

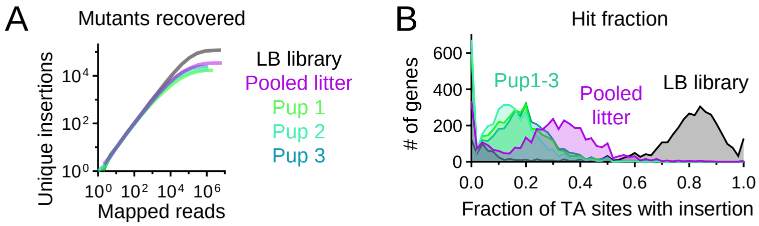

Supplemental figure 1 | Pooling a litter of CD1 pups increases the fraction of the transposon library recovered after infection. Extended data from Fig 1. P4 CD1 mice were intragastrically inoculated with ~5x10^7 CFU of a mariner transposon library. 18-hours later, *V. cholerae* from the SIs of individual pups or the pooled SIs of an entire litter (10 pups) were outgrown overnight on LB, and then sequenced. For comparison, the same library was passaged overnight on LB media (LB library). **A,** The reads from each library were randomly sampled, and the number of unique insertion sites were plotted to depict diversity across sampling depths. **B,** Histogram of fraction of possible insertion sites in each gene disrupted by a transposon insertion. TA sites are sites with a TA dinucleotide. Mariner transposons primarily integrate at TA sites, and data throughout the manuscript is filtered only to include transposons integrated at TA sites.

445

bioRxiv preprint doi: https://doi.org/10.1101/2025.02.12.637895; this version posted February 13, 2025. The copyright holder for this preprint (which was not certified by peer review) is the author/funder, who has granted bioRxiv a license to display the preprint in perpetuity. It is made available under aCC-BY 4.0 International license.

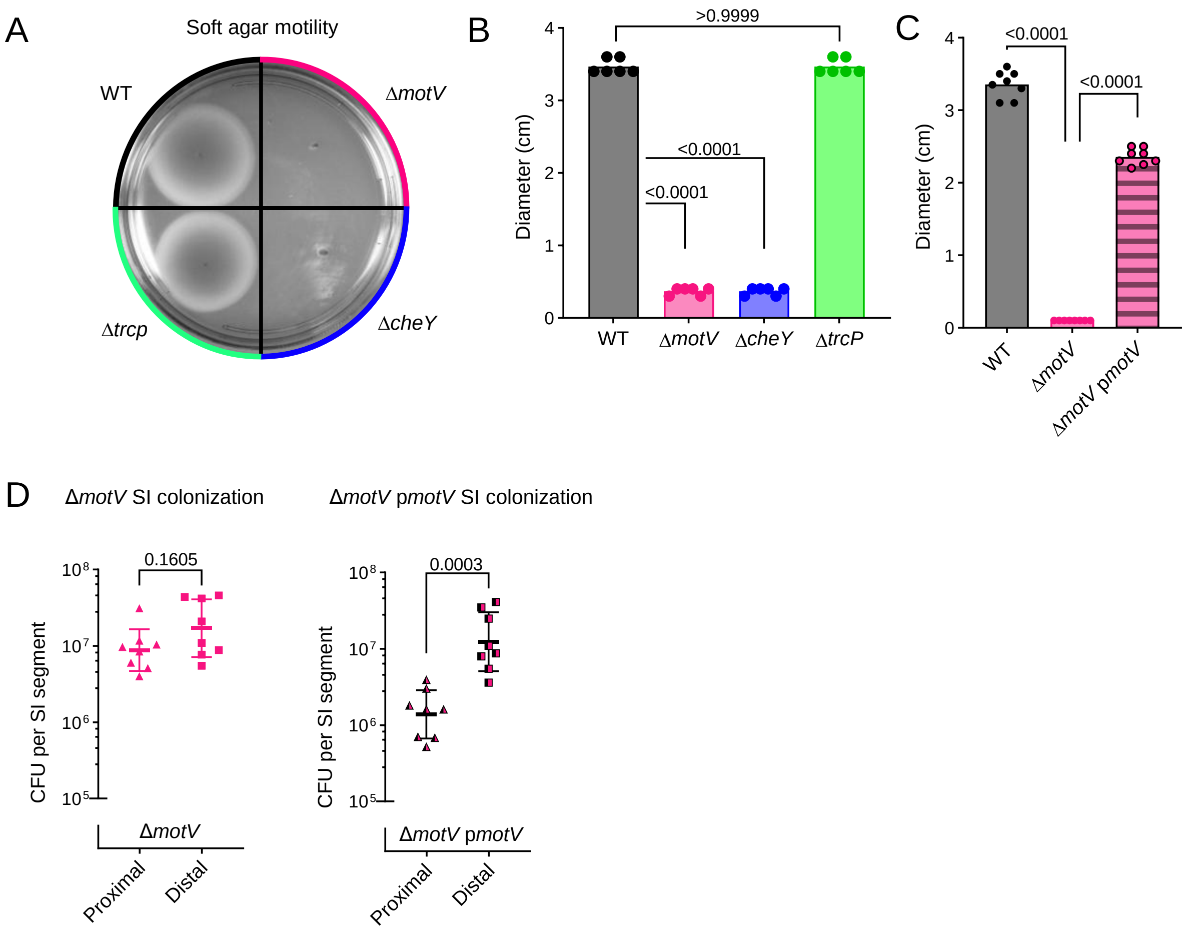

Supplemental figure 2 | Extended observations and complementation of the Δ*motV* mutant. Extended data related to Fig 2. **A-C,** Swarming motility of indicated *V. cholerae* strains in soft agar plates. **A,** Image of a representative plate. **B-C,** Diameter of motility zones. A Δ*trcp* mutant is included as an additional control. Complementation of the Δ*motV* mutant with a plasmid-borne copy of *motV* (p*motV*) restores motility in soft agar. Mean and individual replicates. One-way ANOVA with Dunnett's multiple comparison correction. **D,** CD1 mice were intragastrically inoculated with the Δ*motV* mutant or Δ*motV* complemented with p*motV*, and 18-hours later CFU was enumerated from the indicated SI segments. Geometric mean and standard deviation. Mann-Whitney test.

bioRxiv preprint doi: https://doi.org/10.1101/2025.02.12.637895; this version posted February 13, 2025. The copyright holder for this preprint (which was not certified by peer review) is the author/funder, who has granted bioRxiv a license to display the preprint in perpetuity. It is made available under aCC-BY 4.0 International license.

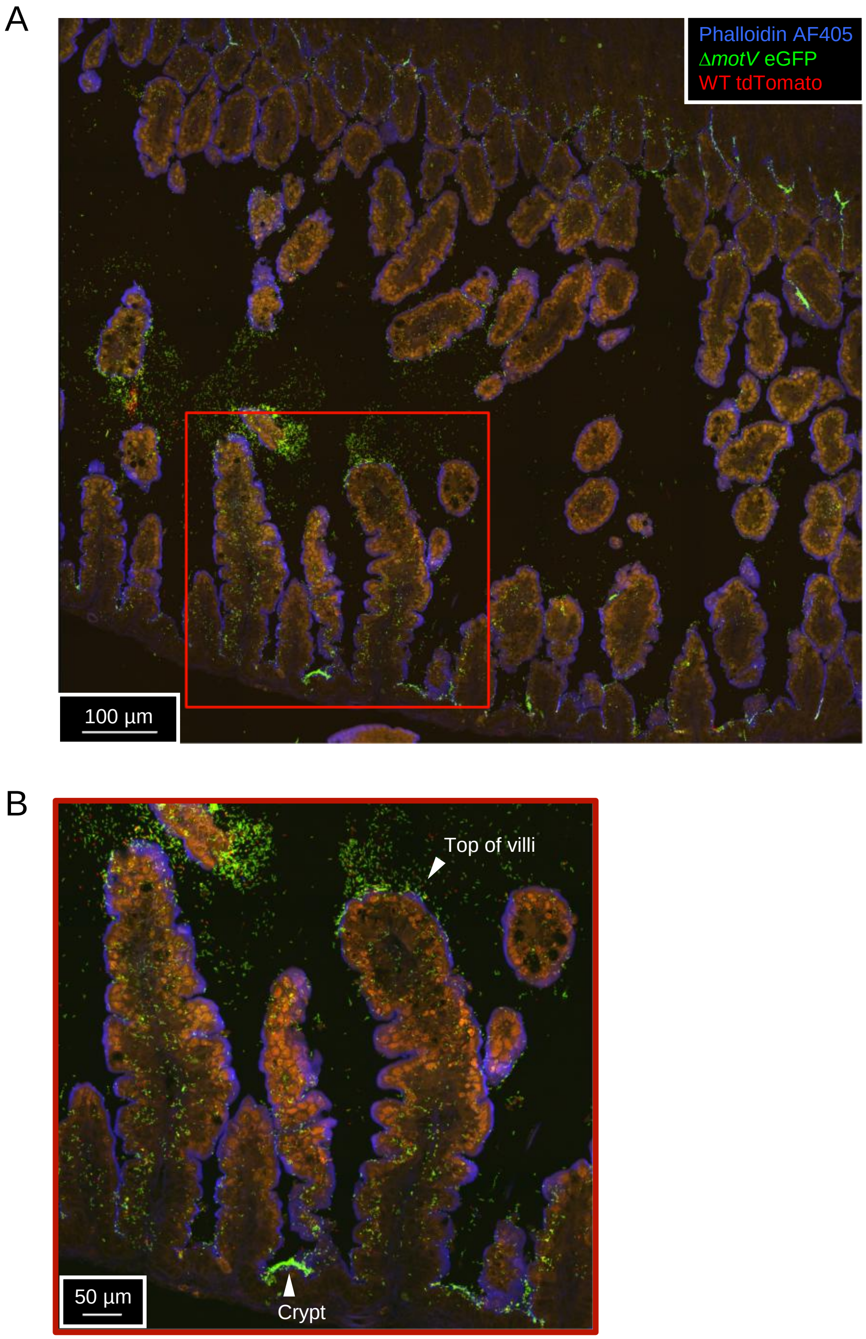

Supplemental figure 3 | Large image of *V. cholerae* localization in the SI. Related to Fig 2. **A,** Large image of a 10 µm cryosection of the medial SI 18-hours after inoculation with ~2 x 10^6 CFU of a 1:1 ratio of WT *lacZ::tdTomato V. cholereae* (red) and Δ*motV lacZ::eGFP V. cholerae* (green). Shown are the villi of the SI on either side of the lumen. Sections were stained with AF405-conjugated phalloidin (blue) and imaged on a spinning disk confocal microscope. **B,** Zoomed-in portion of **A** (400 x 400 µm) showing the top of villi and crypts of the SI in more detail; outlined in red on **A**.

bioRxiv preprint doi: https://doi.org/10.1101/2025.02.12.637895; this version posted February 13, 2025. The copyright holder for this preprint (which was not certified by peer review) is the author/funder, who has granted bioRxiv a license to display the preprint in perpetuity. It is made available under aCC-BY 4.0 International license.

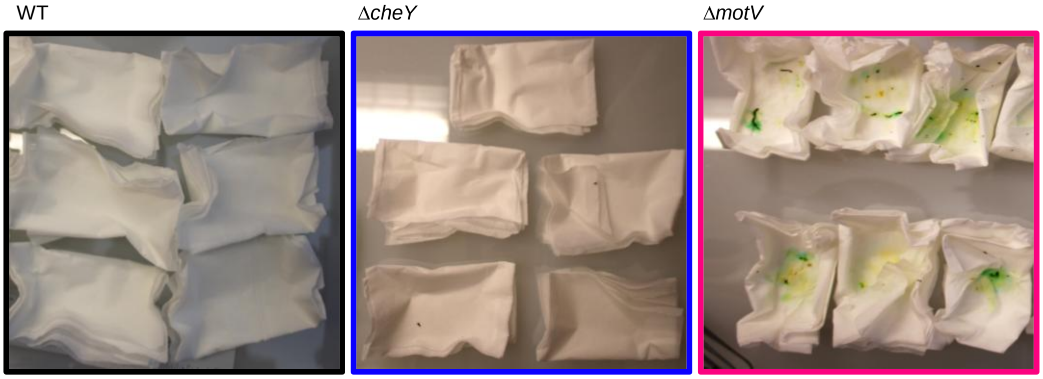

Supplemental figure 4 | Deletion of *motV* increased infant mouse diarrheal discharge during infection with *V. cholerae*. CD1 pups were intragastrically inoculated with the indicated *V. cholerae* strains and individually housed for 18-hours in the pictured bedding. Each tissue is the bedding of a single infected animal. The *V. cholerae* inoculum is dyed green to assist with intragastric gavage, and subsequent discharge of the dye stains the bedding, assisting in visualizing diarrhea discharge.

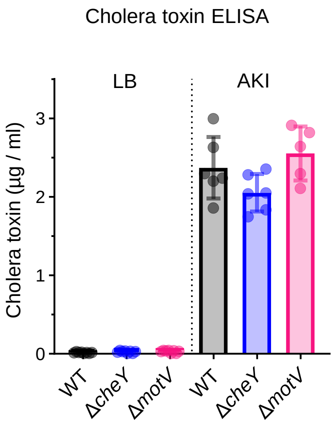

Supplemental figure 5 | *motV* deletion does not change cholera toxin production in culture. ELISA measurement of cholera toxin following culture. Mean and standard deviation. Not significant by one-way ANOVA with Turkey's multiple comparison correction.

bioRxiv preprint doi: https://doi.org/10.1101/2025.02.12.637895; this version posted February 13, 2025. The copyright holder for this preprint (which was not certified by peer review) is the author/funder, who has granted bioRxiv a license to display the preprint in perpetuity. It is made available under aCC-BY 4.0 International license.

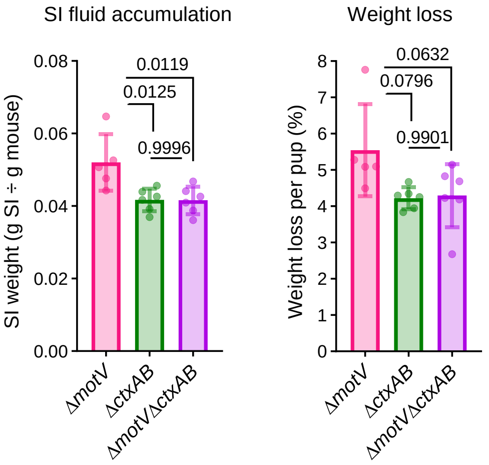

Supplemental figure 6 | Deletion of *ctxAB* decreases the diarrheaogenicity of the Δ*motV* mutant. CD1 pups were intragastrically inoculated with the indicated *V. cholerae* strains and individually housed for 18-hours. Fluid accumulation in the SI was measured by comparing the weight of the animal to the weight of the SI. Weight loss was determined by comparing animal weight before and after infection. Mean and standard deviation. One-way ANOVA with Turkey's multiple comparison correction.
